# Prevalence and Factors Associated With Hepatitis C in Danish Prisons, 2022–2024: A Multicentre Cross‐Sectional Study

**DOI:** 10.1111/jvh.70154

**Published:** 2026-03-02

**Authors:** Jonas Demant, Jacob Søholm, Jeffrey V. Lazarus, Louise Krohn‐Dehli, Christina Egelund, Søren Madsen, Toke Barfod, Cécile Kremer, Stephen Strunge Nilsson, Ditte Andersen Skovdal, Nina Weis

**Affiliations:** ^1^ Department of Infectious Diseases Copenhagen University Hospital Hvidovre Denmark; ^2^ Department of Infectious Diseases Odense University Hospital Odense Denmark; ^3^ Barcelona Institute for Global Health (ISGlobal) University of Barcelona Barcelona Spain; ^4^ CUNY, Graduate School of Public Health and Health Policy New York New York USA; ^5^ Department of Internal Medicine and Infectious Diseases Zealand University Hospital Roskilde Denmark; ^6^ Department of Medical Gastroenterology Zealand University Hospital Køge Denmark; ^7^ Department of Clinical Medicine, Faculty of Health and Medical Sciences University of Copenhagen Copenhagen Denmark; ^8^ Data Science Institute, I‐BioStat, Universiteit Hasselt Hasselt Belgium; ^9^ Department of Clinical Microbiology Copenhagen University Hospital Hvidovre Denmark; ^10^ Department of Clinical Microbiology Copenhagen University Hospital Herlev Denmark; ^11^ Health Team, Danish Prison and Probation Service København Ø Denmark

**Keywords:** cross‐sectional studies, Denmark, hepatitis c, people who use drugs, prisons

## Abstract

Prisons offer a critical opportunity for hepatitis C virus (HCV) elimination, yet current data from Danish correctional facilities are sparse. We conducted a cross‐sectional study in 16 prisons across Eastern Denmark between October 2022 and August 2024, enrolling 651 incarcerated individuals. All participants underwent HCV antibody and RNA testing using dried blood spots and completed a bio‐behavioural risk survey. The prevalence of HCV antibody and HCV RNA was 4.2% (*n* = 26) and 2.0% (*n* = 13) respectively. HCV exposure was most prevalent among individuals with a history of injecting drug use (56.4%), women (11.3%) and foreign‐born individuals (6.7%). In multivariable logistic regression, HCV exposure was significantly associated with injecting drug use (adjusted odds ratio [aOR] 209.11, 95% confidence interval [CI]: 36.16–1209.27), female sex (male vs. female: aOR 0.18, 95% CI: 0.05–0.60) and being born in a low‐prevalence country (aOR 6.22, 95% CI: 1.64–23.61). We observed substantial site‐level variation and care gaps that disproportionately affect marginalised groups. These findings support the implementation of targeted HCV screening at prison intake, along with facility‐specific and population‐tailored interventions, as essential strategies for achieving Denmark's commitment to the World Health Organization's HCV elimination goal.

Hepatitis C is a liver infection caused by the bloodborne hepatitis C virus (HCV). Around 25% of individuals with acute infection spontaneously clear the virus, but persistent infection beyond 6 months typically results in chronic disease [1]. Most people with chronic HCV remain asymptomatic for years, often diagnosed only after significant liver damage [1, 2]. In 2022, an estimated 50 million people (0.7%) were living with HCV, including 8.6 million (0.6%) in Europe [3]. HCV disproportionately affects marginalised populations, particularly people who inject drugs (PWID), who face elevated transmission risks and barriers to testing and treatment [4, 5, 6].

Since 2013, the introduction of oral direct‐acting antivirals (DAAs) has transformed HCV care, offering cure rates exceeding 95% with minimal side effects [7]. In response, the World Health Organization (WHO) set a target to eliminate HCV as a public health threat by 2030 [8, 9]. However, progress remains uneven due to stigma, limited healthcare access and challenges engaging marginalised groups [10].

PWID are central to HCV transmission in high‐income countries, where injecting is the main mode of spread [4, 11]. Structural barriers such as homelessness, mental illness and fragmented care contribute to poor testing and treatment uptake [12, 13]. Decentralised care models in community settings have proven more effective than traditional healthcare approaches [14, 15]. In line with this, WHO recommends expanding HCV screening to correctional settings, including universal testing at prison intake [16].

Prisons offer a strategic setting for HCV elimination. Incarcerated populations bear a disproportionately high burden of infection, largely due to overlapping histories of drug use and imprisonment. Globally, 58% of PWID report prior incarceration [17], and HCV prevalence in prison populations reaches up to 56% globally and 45% in Europe [18, 19]. In Denmark, the most recent prison‐based estimate was 4.2%, a marked decline from 28.9% in 1997 but still nearly 20 times higher than in the general population (0.21%) [20, 21].

Despite this, Denmark lacks a coordinated national strategy for prison‐based HCV screening. Most existing data were collected before 2018, when treatment access expanded following the removal of DAA restrictions [22]. Earlier studies also excluded non‐Danish citizens, approximately 25% of the Danish prison population, who are a priority group for surveillance and care in Europe [23, 24]. In addition, prior research focused only on Western Denmark, omitting the Capital and Zealand regions, where foreign‐born individuals and PWID are more concentrated, and provided limited data on remand facilities [25, 26].

To address these gaps, we conducted a cross‐sectional study in 16 prisons in Eastern Denmark. We aimed to: (1) estimate the prevalence of HCV in prisons in Eastern Denmark; (2) assess demographic and behavioural factors associated with HCV exposure and (3) evaluate self‐reported HCV awareness, testing uptake and treatment history to inform targeted elimination strategies.

## 1 Material and Methods

### 1.1 Study Design and Setting

We conducted an observational cross‐sectional study to estimate the prevalence of HCV exposure and associated risk factors among incarcerated individuals in Eastern Denmark. Data were collected between October 2022 and August 2024 across 16 prison facilities in the Capital and Zealand regions, comprising approximately 1464 incarcerated individuals and accounting for 36% of the national prison population.

Larger facilities were visited repeatedly during the study period to ensure adequate population coverage, whereas smaller facilities were typically visited once. Participants were included only once, and procedures were implemented to avoid duplicate inclusion, including in cases of transfer between facilities. No changes in prison health care provision or HCV‐related interventions occurred during the study period, and the data were therefore pooled for analysis.

The participating institutions included four long‐term prisons and 12 pretrial detention centres, selected due to limited existing HCV data and a high concentration of at‐risk populations. Facility capacities ranged from 15 to 469 individuals and included various security levels: open, semi‐open and high security. Two facilities housed only women, one long‐term prison and one pretrial centre, while the others were male‐only, although three housed a limited number of women.

### 1.2 Participants

Eligible participants were aged ≥ 18 years, either sentenced or in pretrial detention, and able to provide written informed consent. Individuals were excluded if they did not understand Danish or English or if cognitive impairment precluded survey participation.

### 1.3 Sample Size Calculation

A priori sample size estimation aimed to detect an HCV antibody prevalence of 7.4% (based on historical Danish data) with ±2% precision at 95% confidence. Based on the total prison population of 1464 individuals, we estimated that a sample of approximately 455 would be sufficient, using a finite population correction for approximation. However, the study used convenience sampling, and this calculation does not imply a formal random sampling design.

### 1.4 Ethical Approval and Consent

Recruitment was facilitated by posters and prison staff providing information about participation. Participants with scheduled work received compensation to avoid financial loss. The study was conducted following the Declaration of Helsinki. All participants received written and verbal information and provided written informed consent before participation. The Danish Data Protection Agency approved the study, and the Health Research Ethics Committee of Denmark (case number H‐21014740, dated 28 February 2022) confirmed that ethical approval was not required.

### 1.5 Data Collection and HCV Testing

Screening and data collection were performed on‐site by a PhD researcher and a study nurse using convenience sampling. All incarcerated individuals present on screening days were invited to participate. An interviewer‐administered bio‐behavioural survey, available in Danish and English, collected sociodemographic data, HCV testing and treatment history, injecting practices, tattooing, opioid agonist therapy (OAT), incarceration history and perceived HCV status.

Recruitment procedures varied by facility. In lower‐security prisons, staff had unrestricted access to housing areas; in high‐security units, access was restricted and escorted. In two facilities, prison staff invited individuals to a designated testing room.

In larger facilities, multiple screening sessions were conducted to ensure sufficient coverage. To avoid duplicate participation, we maintained screening logs including participant name, date of birth and prison ID number. These were cross‐checked across facilities and visits to ensure each individual was included only once.

All participants underwent rapid HCV antibody testing via finger‐prick (In‐Tech, Xiamen, China). Dried blood spots (DBS) were collected from all participants for HCV RNA analysis, regardless of antibody result. Optional HBV and HIV testing was offered using DBS. Blood was collected with a sterile, button‐activated lancet (Accu‐Chek Safe‐T‐Pro Plus, Roche, Basel, Switzerland), and DBS were spotted on Whatman cards (Merck, Darmstadt, Germany). Participants with positive antibody results were offered on‐site liver elastography using a mobile FibroScan 402 (Echosens). Samples were analysed at the Department of Clinical Microbiology, Copenhagen University Hospital, Hvidovre. Detailed laboratory methods have been published previously [27].

### 1.6 Bias and Representativeness

As this was a convenience sample based on voluntary participation, selection bias could not be excluded. To assess representativeness, participant characteristics (age, sex, foreign‐born status and citizenship) were compared descriptively to the national prison population using data from the Danish Correctional Services' annual report [28].

### 1.7 Follow‐Up

Results from HCV rapid antibody tests were available within 10 min, while HCV RNA, HIV and HBV results were typically returned within 10 days. All results were documented in participants' electronic prison medical records. In low‐security facilities, individuals accessed records directly; in high‐security prisons, healthcare staff facilitated access.

Participants with detectable HCV RNA, HIV or HBV were informed in a private consultation and offered counselling and treatment. Interpreters were provided when needed. All HCV RNA‐positive participants were eligible for treatment, regardless of legal or migration status.

An infectious disease specialist at the regional hospital prescribed DAA treatment. Pan‐genotypic regimens, glecaprevir/pibrentasvir or sofosbuvir/velpatasavir, were used per Danish national guidelines [29], eliminating genotype testing and enabling faster initiation. Medications were dispensed through prison healthcare units. Follow‐up tests were conducted at treatment completion and 12 weeks posttreatment.

### 1.8 Outcomes

The primary outcomes were the prevalence of HCV antibody positivity (i.e., a reactive result on a qualitative rapid antibody test) and detectable HCV RNA, defined as a viral load above the DBS assay's 95% limit of detection (≥ 525 IU/mL). Secondary outcomes included factors associated with HCV exposure, defined as positivity for either HCV antibody or HCV RNA. Additional outcomes included the accuracy of self‐reported HCV status and historical access to testing and treatment among key subgroups.

### 1.9 Variables

Survey‐based variables included age, sex, drug use (type and route), tattooing, OAT status, country of birth, incarceration history, facility type, prior HCV testing and perceived HCV status.

### 1.10 Statistical Analyses

HCV prevalence was expressed as proportions with 95% confidence intervals (CI). Generalised linear mixed models (GLMMs) were used to assess associations between factors and HCV exposure (antibody or RNA positivity), accounting for clustering by facility through random intercepts. Although sampling was not random, clustering by facility was accounted for in the regression analyses using GLMMs.

Candidate variables with *p* < 0.150 in univariate models and clinical relevance were entered into a multivariable GLMM. Backward stepwise selection was used to retain only variables with *p* < 0.05. Adjusted odds ratios (aOR) and 95% CI are reported. In model building, complete cases for the included variables are used. Missing responses for the drug use variable were handled by including a separate ‘unknown’ category in the multivariable model, allowing individuals with missing information to be retained in the analysis. Descriptive statistics were used to evaluate self‐reported HCV status, including sensitivity, positive predictive value (PPV) and negative predictive value (NPV). All analyses were performed using R version 4.4.3 (R Core Team, 2022).

## 2 Results

### 2.1 Eligibility and Participation

Across 16 prisons in Eastern Denmark, 651 individuals participated, representing 45% of the incarcerated population (*n* = 1464) in these facilities. Participation ranged from 21% to 73% in sentenced populations and from 25% to 83% in pretrial populations. Main reasons for nonparticipation were absence on the screening day, language barriers and refusal.

The study sample mirrored the national prison population in age, country of birth and sex: 29.9% of participants were foreign‐born (vs. 31.5% nationally), median age was 35 years (vs. 34) and 85.0% were men (vs. 90.5%). The proportion with Danish citizenship was also comparable (75.3% vs. 73.5%), and the share without Danish citizenship or a personal identification number was 7.5% (49 participants) in our study, versus 10% nationally.

### 2.2 Drug Use Characteristics

Among 600 participants who responded to drug use questions, 404 (67.3%) reported lifetime drug use, and 39 (6.5%) had ever injected. Lifetime injecting drug use prevalence was higher among women (12.4%) than men (5.1%). Of those who had injected, 33.3% reported sharing equipment. One participant reported injecting during current incarceration. Among the 29 who provided the time of last injection, the median was 4 years before imprisonment (IQR: 1.3–9.8).

Among those with lifetime drug use, 382 (94.6%) reported stimulant use, followed by 102 (25.2%) for opioids and 81 (20.0%) for other substances. When asked about the drug used most frequently, 316 (89.5%) identified stimulants, while 32 (9.1%) cited opioids. Notably, half of those who named opioids as their primary drug described oral formulations (e.g., tramadol), which are not typically associated with injecting‐related HCV risk. Regarding OAT, 6.6% (*n* = 32) were currently receiving treatment, and 5.8% (*n* = 28) reported prior OAT (Table 1).

**TABLE 1:** Sociodemographic and behavioural characteristics of the study population (*n* = 651) recruited from 16 prisons in Denmark, 2022–2024.

Sociodemographic characteristics	Total population (*n* = 651)	HCV‐exposed (*n* = 27)
	*n* (%)	*n* (% of HCV‐exposed)
Age (*n* = 651)		
Age, years [median, IQR]	35 [28;45]	41 [34.3;47.5]
Gender (*n* = 646)		
Female	97 (15.1)	11 (40.7)
Male	549 (84.9)	16 (59.3)
Birth country (*n* = 647)		
Denmark	453 (70.0)	14 (51.9)
Other		
Low‐prevalence country	115 (17.8)	10 (37.0)
Moderate to high prevalence country	79 (12.2)	3 (11.1)
Citizenship (*n* = 647)		
Danish	487 (75.3)	18 (66.7)
Other	160 (24.7)	9 (33.3)
Prison type (*n* = 651)		
Long‐term	175 (26.9)	12 (44.4)
Pretrial (remand)	476 (72.1)	15 (55.6)
Lifetime years in prison (*n* = 540)		
< 10 years	491 (90.9)	15 (55.6)
≥ 10 years	49 (9.1)	6 (22.2)
Mental health and housing history		
History of being unhoused (*n* = 600)	168 (28.0)	16 (59.3)
History of mental illness (*n* = 595)	157 (26.8)	12 (44.4)
Drug use characteristics		
Ever used drugs (*n* = 600)	404 (67.3)	24 (88.9)
Drug use unknown	50 (7.9)	1 (3.7)
Ever injected drugs	39 (6.5)	22 (81.5)
Ever shared materials for injecting drugs (*n* = 39)	13 (33.3)	10 (37.0)
Drugs used (*n* = 404)		
Opioids (heroin, tramadol)	102 (25.3)	22 (81.5)
Stimulants (cocaine, amphetamine)	382 (94.6)	24 (88.9)
Other (MDMA, GHB, benzos, other)	81 (20.1)	3 (11.1)
Drugs mainly used (*n* = 353)		
Opioids	32 (9.1)	9 (33.3)
Stimulants	316 (89.5)	14 (51.9)
Other	5 (1.4)	0 (0.0)
Tattoos (*n* = 603)		
No tattoos	219 (36.3)	4 (14.8)
Potentially nonsterile	120 (19.9)	8 (29.6)
Safe	239 (39.6)	10 (37.0)
Enrolled in opioid antagonist therapy (*n* = 483)		10 (37.0)
Yes	32 (6.6)	13 (48.1)
No, never	422 (87.4)	7 (25.9)
Not currently, but in the past	28 (6.0)	5 (18.5)
HCV status and history of test and treatment (*n* = 651)		
HCV antibody detected	26 (3.9)	n.a.
HCV RNA detected	13 (2.0)	n.a.
Previously tested for HCV (self‐reported)	141 (21.7)	18 (66.7)
Previously treated for HCV (self‐reported)	9 (1.4)	7 (25.9)

*Note:* IDU was assessed among participants who responded to the lifetime drug use question (*n* = 600). Drug categories are not mutually exclusive. ‘Opioids’ include heroin, tramadol, morphine, etc.; ‘Stimulants’ include cocaine and amphetamine; ‘Other’ includes MDMA, GHB and benzodiazepines. ‘Drugs mainly used’ reflects the substance the participant reported using most frequently. Tattoo setting categorised as ‘safe’ (professionally administered) or ‘potentially non‐sterile’ (e.g., in prison). Responses marked ‘Don't know’ or missing were excluded; totals may therefore not sum to 651 across all variables.

Abbreviations: HCV Ab, hepatitis C virus antibody; HCV RNA, HCV ribonucleic acid (viraemia); IDU, injecting drug use.

### 2.3 HCV, HIV and HBV Prevalence

**FIGURE 1:**
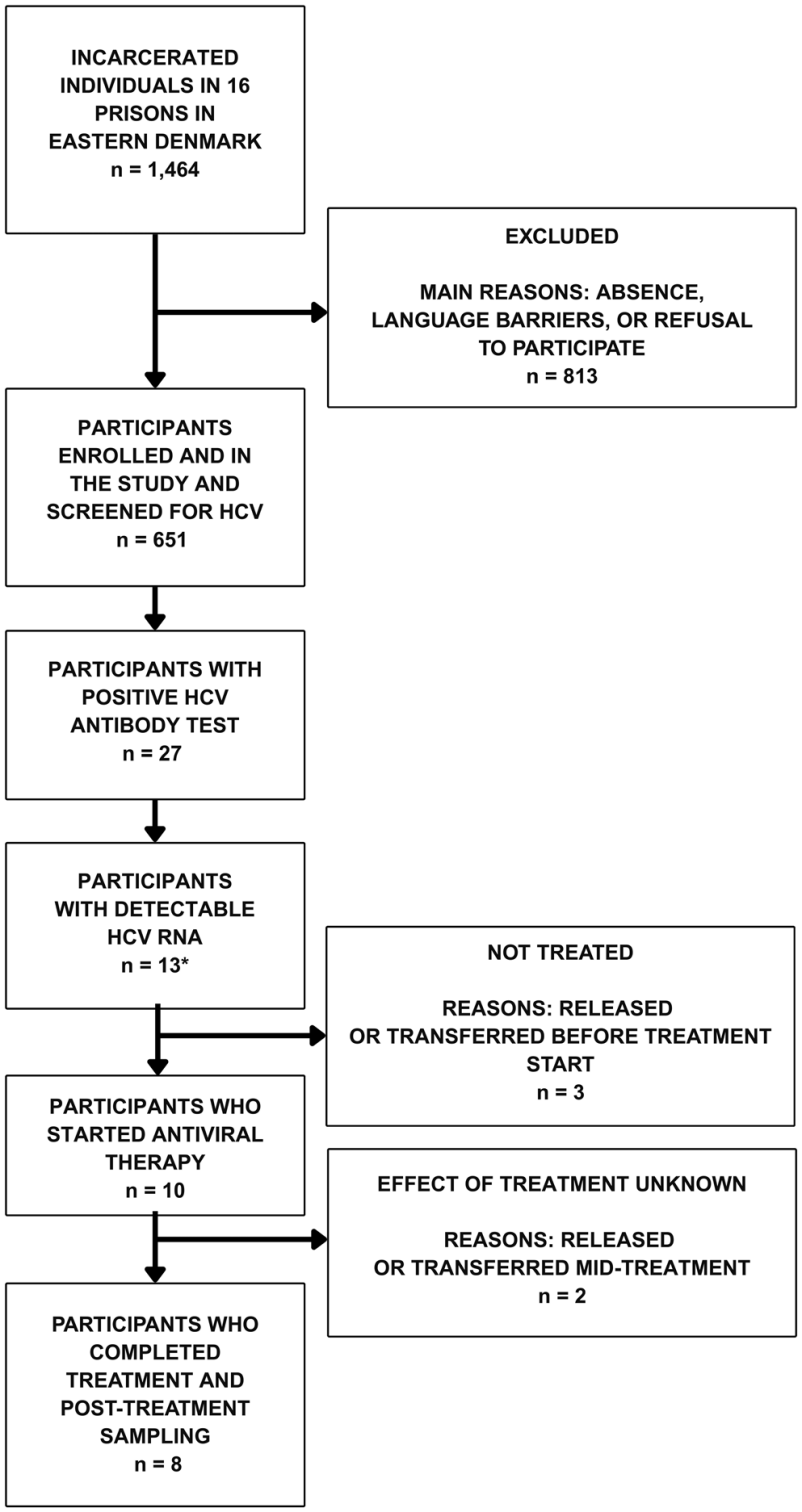
Flow diagram of participant inclusion, testing and treatment for hepatitis C virus among 651 incarcerated individuals across 16 prisons in Denmark, 2022–2024. One participant had HCV RNA detected but not HCV antibodies. HCV, hepatitis C virus; Antiviral Therapy: direct‐acting antiviral agents.

A total of 26 participants (4.0%, 95% CI: 2.7%–5.9%) tested positive for HCV antibodies, and 13 (2.0%, 95% CI: 1.1%–3.5%) had detectable HCV RNA. One individual was RNA‐positive but antibody‐negative, possibly indicating acute infection. In total, 27 participants (4.2%, 95% CI: 2.8%–6.1%) were classified as HCV‐exposed, defined as antibody‐ or RNA‐positive. Participant flow through eligibility, testing and treatment is shown in Figure 1.

HCV exposure was most prevalent among individuals with a history of injecting drug use (22/39; 56.4%), particularly those who reported sharing equipment (10/13; 76.9%). Women had a higher exposure prevalence than men (11.3% vs. 2.9%). Among foreign‐born participants, 13 of 194 (6.7%) were exposed, compared to 14 of 455 (3.1%) among Danish‐born. Among non‐Danish citizens without a personal identification number, six of 49 (12.5%) were HCV‐exposed.

No HCV‐exposed individuals were identified in seven of the 16 prisons, all small pretrial detention. Of the 27 exposed participants, 12 (44.4%) were incarcerated in long‐term facilities and 15 (55.6%) in pretrial centres. Active HCV infections (RNA‐positive) were detected across six prisons, including two long‐term and four pretrial institutions. The highest HCV exposure prevalence was in the women's facility, where 11 individuals (12.3%) were exposed and five had active infection.

HIV was detected in four participants (0.6%), all previously diagnosed and receiving treatment. HBV was detected in one participant (0.15%) who was released and deported prior to clinical follow‐up, but was informed of the diagnosis.

### 2.4 Awareness of HCV Status and Accuracy of Self‐Report

In addition to estimating HCV prevalence, we assessed participants' awareness of their HCV status. Of the 26 participants with laboratory‐confirmed HCV exposure, 15 (57.7%) provided a definitive ‘Yes’ or ‘No’ response, while 11 (42.3%) were unsure. Among those with confirmed exposure who gave a definitive response, the sensitivity of self‐reported HCV status was 66.7% (10/15). In the full study population, 72.1% reported being unsure of their status. Among the 15 who gave definitive responses, the positive predictive value (PPV) was 90.9%, and the negative predictive value (NPV) was 96.2%.

### 2.5 Prior Testing and Treatment

Of the total study population, 141 participants (21.7%) reported having previously been tested for HCV. Among those with confirmed HCV exposure, 18 of 27 (66.7%) had undergone prior testing. Prior testing was also reported by 22 of 39 participants (56.4%) with a history of injecting drug use and by 51 of 194 foreign‐born individuals (26.3%). A higher proportion of women reported previous testing compared to men (23 of 62, 37.1% vs. 118 of 628, 18.8%).

Nine participants (1.4%) reported receiving HCV treatment; seven had antibodies and no detectable RNA, consistent with successful treatment, while the remaining two likely misreported their infection history. Among the 13 with active infection, seven (53.8%) had been previously tested, and four (30.8%) were aware of their infection. None of the 13 reported prior treatment.

### 2.6 Factors Associated With HCV Exposure

In multivariable analysis, a history of injecting drug use (IDU) was strongly associated with HCV exposure (adjusted odds ratio [aOR] 209.11; 95% CI: 36.16–1209.27; *p* < 0.001), as was being born in a low‐prevalence country other than Denmark (aOR 6.22; 95% CI: 1.64–23.61; *p* = 0.007). In addition, being male was associated with a lower odds of HCV exposure (aOR 0.18; 95% CI: 0.05–0.60; *p* = 0.005). These estimates reflect adjustment for other covariates, including age, tattoos and drug use history. Full univariate results are presented in Table 2. Due to missing data, lifetime years in prison were excluded from the final model, though a sensitivity analysis including it did not change the results.

**TABLE 2:** Univariate and multivariable associations with HCV exposure among incarcerated individuals in 16 prisons, Denmark, 2022–2024 (*n* = 651).

Characteristics	Univariate		Multivariable	
	OR (95% CI)	*p*	AOR (95% CI)	*p*
Age (*n* = 641)	1.03 (0.99–1.06)	0.064		
Sex (*n* = 646)				
Female	1 (REF)			
Male	0.24 (0.11–0.81)	< 0.001	**0.18** (**0.05–0.60**)	0.005
Prison type (*n* = 651)	1 (REF)			
Long‐term				
Pretrial	0.62 (0.23–1.82)	0.37		
Lifetime years in prison (*n* = 540)				
≤ 10 years	1 (REF)			
10+ years	6.49 (1.96–21.49)	0.002		
Birth country (*n* = 647)				
Denmark	1 (REF)			
Low‐prevalence country	3.22 (1.32–7.66)	0.009	**6.22** (**1.64**–**23.61**)	0.007
Moderate‐to‐high‐prevalence country	1.40 (0.31–4.61)	0.611	2.90 (0.41–20.59)	0.286
Citizenship (*n* = 647)				
Other/non‐Danish	1 (REF)			
Danish	0.51 (0.21–1.28)	0.14		
Tattoo (*n* = 603)				
No tattoos	1 (REF)			
Safe	2.20 (0.73–8.07)	0.19		
Potentially nonsterile	3.49 (1.01–13.80)	0.053		
Ever used drugs (*n* = 600)				
No	1 (REF)			
Yes	8.16 (2.30–52.23)	0.01		
Drugs used (*n* = 598)				
Opioids	1 (REF)			
Stimulants	0.11 (0.01–2.43)	0.077		
Poly use	0.72 (0.11–14.30)	0.771		
Injecting drug use (*n* = 404)				
Never	1 (REF)			
Ever	308.32 (69.12–3050.95)	< 0.001		
Drug use (*n* = 600)				
No drug use	1 (REF)			
Noninjecting drug use	0.57 (0.07–4.94)	0.585	1.15 (0.15–8.97)	0.891
Injecting drug use	171.85 (39.17–1345.46)	< 0.001	**209.11** (**36.16**–**1209.27**)	**< 0.001**
Unknown	2.45 (0.11–28.77)	0.484	**1.96** (**0.17–23.32**)	**0.594**
Ever shared materials for IDU (*n* = 39)				
No	1 (REF)			
Yes	4.27 (0.81–32.45)	0.106		
Ever opioid antagonist therapy (*n* = 482)				
Yes	1 (REF)			
No, never	0.32 (0.07–1.19)	0.01		
Not currently, but in the past	0.02 (0.004–0.06)	< 0.001		

*Note:* All estimates are from generalised linear mixed models (GLMMs) with a logit link and a random intercept for prison to account for clustering. Birth country was categorised based on national HCV prevalence: low (< 1%) vs. moderate‐to‐high (≥ 1%). Drug use was classified as none, noninjecting (e.g., oral or nasal administration) or injecting. Tattoo setting was categorised as ‘safe’ (professionally administered) or ‘potentially non‐sterile’ (e.g., in prison). Sharing of IDU materials was assessed only among participants reporting lifetime injecting. Due to missing data, denominators vary slightly between variables. Only variables with *p* < 0.05 were retained in the multivariable model. In the multivariable model, individuals with missing responses to the drug use question were retained via inclusion of a separate ‘Unknown’ category.

Abbreviations: AOR, Adjusted odds ratio; CI, Confidence interval; HCV, Hepatitis C virus; IDU, Injecting drug use; OR, Odds ratio.

### 2.7 Follow‐Up and Treatment Outcomes

**FIGURE 2:**
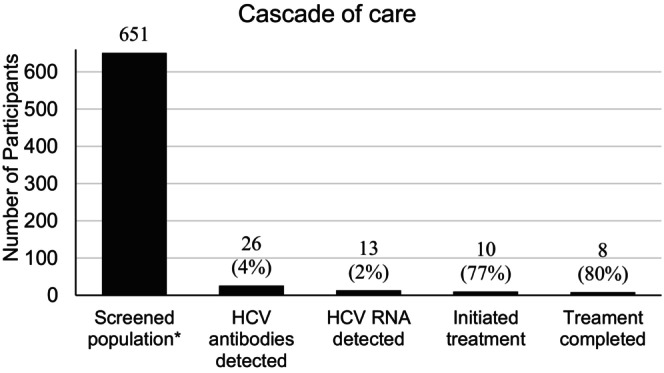
Cascade of care for 651 incarcerated individuals screened across 16 Danish prisons, 2022–2024. The screened group represents 45% of the total incarcerated population (*n* = 1464) in the included facilities.

Of the 13 participants with detectable HCV RNA, 10 initiated treatment with DAAs. Eight patients completed treatment and achieved undetectable HCV RNA. Two were released or transferred before treatment initiation: one was transferred without documented follow‐up, and one was deported with medication but without confirmed treatment completion. The remaining three did not initiate treatment during the study period. The median time from DBS testing to treatment initiation was 47 days (IQR: 29–178). The cascade of care is shown in Figure 2.

## 3 Discussion

In this multicentre cross‐sectional study conducted across prisons in Eastern Denmark, 2.0% of incarcerated individuals had detectable HCV RNA, consistent with active infection. Injectable drug use was strongly associated with HCV exposure, although estimates were limited by the small number of exposed participants.

The observed HCV prevalence in this study lies at the lower end of estimates reported in prison populations globally (HCV Ab: 0.3%–74.4%; HCV RNA: 0%–56.3%) and in Europe (HCV Ab: 0.5%–74.4%; HCV RNA: 0%–45.0%) [18]. Many estimates come from single‐centre studies and lack national representativeness, but even compared with multicentre studies from Belgium, England, Norway and Sweden, our findings remain at the low end (HCV Ab: 5.0%–24.5%) [29, 30, 31, 32]. In Denmark, this reflects a long‐term decline in prison HCV prevalence—from 28.9% active infection in 1997 to 2.0% in this study [33]. Similar declines have been observed in countries like Italy and England, where national strategies, including systematic prison‐based screening and treatment, have contributed [31, 34]. Denmark, by contrast, has no coordinated prison HCV strategy, suggesting the decline may be driven by indirect effects of broader national measures, such as lifting treatment restrictions in 2018 and expanding community‐based outreach targeting high‐risk populations [35, 36, 37, 38, 39].

The decline may also reflect broad opioid agonist therapy (OAT) availability in Denmark, which reduces HCV transmission and supports direct‐acting antiviral (DAA) treatment engagement [40, 41, 42]. OAT coverage is relatively high in both community and prison settings [43, 44], and average dosing levels exceed those reported in many other European countries [44, 45]. However, sustained access to OAT during incarceration and after release remains essential, particularly given the elevated risk of HCV transmission in the postrelease period [46].

Another contributor to the decline in HCV prevalence may be the changing epidemiology of injection drug use in Denmark. People who inject drugs (PWID) now represent an aging population, with few younger individuals initiating injection. Surveillance data show new HCV infections are increasingly rare among those under 45, while the mean age of the Danish prison population is 34 years [22, 47]. Although the overlap between injecting and incarceration is well known, it appears to be declining in Denmark. Prison‐based studies over three decades show a steady decline in the proportion of incarcerated PWID: from 43% in 1997 [33] to 8.5% in 2018 [20] and 6.5% in this study. This is notably lower than the estimated 21% lifetime IDU prevalence in European prisons [48] and may also reflect high OAT coverage linked to reduced criminal activity [49].

Although HCV prevalence has declined in Danish prisons, a residual burden remains, concentrated in a highly marginalised subset. Among the 27 individuals with confirmed HCV exposure, 13 had a history of unstable housing, 12 reported mental illness, 13 were foreign‐born and 22 had injected drugs (48%, 44%, 48% and 81% respectively). Despite elevated risk, care gaps persist: nine had never been tested, and nine were unsure. This underscores prisons as key opportunities to reach individuals with poor access to HCV care.

Foreign‐born individuals were prominently represented within this vulnerable subgroup, accounting for nearly half of those with HCV exposure. In total, 6.7% of foreign‐born participants were HCV‐exposed, unchanged from earlier Danish prison studies [20], suggesting they may not have benefited equally from recent declines. Lifetime injecting drug use prevalence was similar between foreign‐born and Danish‐born individuals (6.7% vs. 6.5%), indicating the higher HCV burden is not explained by increased IDU exposure.

Instead, disparities in access to testing and treatment likely contribute to the elevated prevalence in this group. Only 26.3% of foreign‐born participants reported prior testing, and some with active infection believed they were ineligible for treatment. This underscores the need for clear communication about healthcare entitlements and reflects broader European evidence that migrants face linguistic and systemic barriers to HCV care [50, 51, 52]. Notably, the higher prevalence among foreign‐born participants may partly stem from those without Danish citizenship or a personal identification number, who are excluded from the public healthcare system and had twice the HCV exposure rate (12.5%). Given that nearly one‐third of the Danish prison population is foreign‐born, targeted screening and linkage to care remain public health priorities.

The multivariable analysis identified an association between HCV exposure and being born in a low‐prevalence country. This finding should be interpreted with caution due to small numbers and wide confidence intervals. The association remained significant after adjustment for injecting drug use, suggesting that injecting drug use alone does not fully explain the observed pattern. Residual confounding, misclassification of exposures, or unmeasured factors related to migration or incarceration history may therefore contribute. Consistent with previous literature, a large meta‐analysis found that HCV prevalence is generally highest among migrants from higher‐prevalence regions [24], supporting the interpretation that our observed association may be spurious or context‐specific. The adjusted odds ratio for injecting drug use was high, but the wide confidence interval reflects sparse data and statistical uncertainty. While the direction of association aligns with extensive existing evidence, the precise magnitude should be interpreted with caution.

Women were notably overrepresented among individuals with HCV exposure, accounting for 41% of exposed participants despite comprising only 15% of the study sample. This pattern persisted in the multivariable model, where female sex was independently associated with higher odds of HCV exposure. This aligns with Danish cohort studies reporting that women are less likely to access HCV testing and treatment [13, 53], and with international data showing both elevated HCV prevalence and lower DAA treatment initiation among women compared to men [54]. Women who inject drugs also face greater barriers to accessing sterile injection equipment, increasing HCV risk [55]. In this study, women had a higher lifetime prevalence of injecting drug use (12.4% vs. 5.1% in men), consistent with global patterns showing women are more frequently incarcerated for drug‐related offences [56]. These findings underscore the need for gender‐responsive interventions, particularly in women's prison facilities.

Although our screening uptake was comparable to that of similar multicentre studies [32], it remains modest. Universal opt‐out testing improves HCV detection and treatment [57, 58], but modelling studies suggest it is not cost‐effective in lower‐prevalence settings like ours [59]. In such contexts, risk‐based strategies targeting individuals with a history of injecting drug use may be more appropriate [60]. Regardless of strategy scale, timing is critical for prison‐based HCV interventions, and screenings should occur at entry [61]. In Denmark, most prison sentences are under 4 months; sufficient time for diagnosis and 8–12 weeks DAA therapy. Our study found a median delay of 47 days between DBS testing and treatment initiation. This could be reduced through point‐of‐care testing and streamlined linkage [61, 62]. Although we did not directly assess HCV incidence, no participants reported injecting drug use during incarceration, which suggested limited ongoing transmission and supported intake screening as a timely intervention.

Regarding risk‐based strategies, our results indicate that self‐report is not a reliable proxy for HCV status. While the positive and negative predictive values were high among participants who gave definitive answers (PPV 90.9%, NPV 96.2%), sensitivity was only 66.7% and over 70% of participants were unsure of their status. This limits the utility of self‐report and aligns with findings from an Australian prison cohort, where similar discordance and uncertainty were reported [63]. Among those uncertain of their status, risk stratification using indicators such as injecting drug use, OAT enrolment, or incarceration history may help prioritise testing.

To improve case‐finding, risk‐based strategies should be supplemented with awareness‐raising efforts and time‐limited screening campaigns, both shown to enhance testing and treatment uptake [64]. These initiatives could be prioritised in facilities with higher observed case counts, such as the women's prison and certain pretrial centres, where variation may reflect unmeasured risks or distinct profiles. Although immigration detention centres were not included, the disproportionate burden among non‐Danish citizens, along with findings from similar settings, suggests targeted screening may be warranted [65]. Engaging prison staff in planning and implementing screening may further increase participation and operational efficiency [66]. Together, these approaches could help strengthen the role of prison‐based interventions in Denmark's broader HCV elimination strategy.

This study has several strengths. The multicentre design enhances generalisability, with 16 facilities represented, including women's facilities and, for the first time, participants from multiple remand/pretrial centres. Inclusion of Capital and Zealand region sites addressed a key geographic gap. Dried blood spot testing enabled the detection of both past exposure and active infection, and inclusion regardless of legal status allowed a more representative sample.

Several limitations should be acknowledged. Participation was voluntary, and recruitment procedures varied across facilities, introducing potential selection bias. Behavioural data were self‐reported and thus subject to social desirability and recall biases. Language and other barriers may have disproportionately affected individuals without Danish citizenship or a personal ID number, who had higher HCV prevalence and were slightly underrepresented, potentially biasing prevalence estimates downward. In contrast, women were slightly overrepresented and had higher HCV prevalence, possibly inflating overall prevalence. Unweighted prevalence estimates mean facility‐level participation variations may have influenced overall results. In larger facilities, multiple screening visits were conducted to improve coverage. However, not all individuals were necessarily present or reachable during these visits, which may have affected screening uptake and representativeness. Finally, the small number of HCV‐exposed individuals limited power in the multivariable analysis, and wide confidence intervals indicate estimates should be interpreted with caution.

## 4 Conclusion

Our findings suggest that while HCV prevalence in Danish prisons is declining, a residual burden persists, especially among people who inject drugs, foreign‐born individuals and incarcerated women. A combination of risk‐based entry testing, supported by point‐of‐care diagnostics, awareness efforts and active engagement of prison healthcare staff, may offer a feasible and effective approach to reaching these groups and advancing national elimination goals.

## Funding

This work was supported by the Hans and Nora Buchard Foundation (64235), Aage og Johanne Louis‐Hansens Fond (22‐2B‐10046), L.F. Foght Foundation (22.040), Simon Spies Fonden (18‐09‐2023), Lizzi and Mogens Staal Foundation (2021‐0394), Dagmar Marshalls Fond (13‐05‐2024), Hvidovre Hospital (R8‐A170), Department of Clinical Medicine, Faculty of Health and Medical Sciences, University of Copenhagen (2396630/4478), Simon Fougner Hartmann's Family Foundation (2020‐0003) and Instituto de Salud Global de Barcelona (CEX2023‐0001290‐S).

## Conflicts of Interest

J.D. received funding from Gilead Sciences (Grant No. 21671). J.V.L. received grants to his institutions from AbbVie, Boehringer Ingelheim, Echosens, Gilead Sciences, Madrigal Pharmaceuticals, Moderna, MSD, Novo Nordisk, Pfizer and Roche Diagnostics; consulting fees from Echosens, GSK, Madrigal Pharmaceuticals, Novavax, Novo Nordisk and Pfizer; a paid leadership role at the Global NASH Council (ended December 2024) and honoraria for lectures from AbbVie, Echosens, Gilead Sciences, GSK, Janssen, Moderna, MSD, Novo Nordisk, Pfizer and Prosciento, all outside of the submitted work. He also held unpaid leadership roles or board memberships in organisations including Healthy Livers, Healthy Lives, the Global Think‐tank on Steatotic Liver Disease, MASH Cities and HIV Outcomes. The remaining authors declare no conflicts of interest.

## Data Availability

The data that support the findings of this study are available from the corresponding author upon reasonable request.
